# NADPH Oxidase Signaling Pathway Mediates Mesenchymal Stem Cell-Induced Inhibition of Hepatic Stellate Cell Activation

**DOI:** 10.1155/2018/1239143

**Published:** 2018-05-10

**Authors:** Haowen Qiao, Yu Zhou, Xingping Qin, Jing Cheng, Yun He, Yugang Jiang

**Affiliations:** ^1^Department of Physiology, School of Basic Medicine, Wuhan University School of Medicine, 185 Donghu Street, Wuhan 430071, China; ^2^Department of Neurosurgery, The Second Xiangya Hospital of Central South University, Changsha, Hunan 410000, China; ^3^Department of Infectious Diseases, The Third People's Hospital of Shenzhen, No. 29 Bulan Road, Shenzhen 518000, China

## Abstract

**Background:**

Bone marrow-derived mesenchymal stem cells (BMSCs) have blossomed into an effective approach with great potential for the treatment of liver fibrosis. The aim of this study was to investigate the underlying antifibrosis mechanisms by which the BMSC inhibit activated hepatic stellate cells (HSCs) in vivo and in vitro.

**Methods:**

To study the effect of human bone marrow-derived mesenchymal stem cells (hBM-MSCs) on activated HSCs, we used HSCs and the coculture systems to evaluate the inhibition of activated HSCs from the aspects of the apoptosis of activated HSCs. In addition, activation of NADPH oxidase pathway and the changes in liver histopathology were tested by using the carbon tetrachloride- (CCl_4_-) induced liver fibrosis in mice.

**Results:**

Introduction of hBM-MSCs significantly inhibited the proliferation of activated HSCs by inducing the apoptosis process of activated HSCs. The effect of hBM-MSCs reduced the signaling pathway of NADPH oxidase in activated HSCs. Besides, the signaling pathway of NADPH oxidase mediated hBM-MSC upregulation of the expression of the peroxisome proliferator-activated receptor *γ* and downregulation of the expression of *α*1(I) collagen and alpha-smooth muscle actin (*α*-SMA) in activated HSCs. Moreover, the hBM-MSC-induced decrease in the signaling pathway of NADPH oxidase was accompanied by the decrease of the activated HSC number and liver fibrosis in a mouse model of CCl_4_-induced liver fibrosis.

**Conclusion:**

The hBM-MSCs act as a promising drug source against liver fibrosis development with respect to hepatopathy as a therapeutic target.

## 1. Introduction

Liver fibrosis, the result of chronic liver injury caused by various factors, is characterized by overexpression and accumulation of extracellular matrix (ECM) proteins and is associated with the activation of hepatic stellate cells (HSCs) [1, 2]. Since activated HSCs are the major collagen-producing cells in liver injury and the activation of HSCs is considered to be a crucial step in the development of liver fibrosis [3–5], thus, it is essential and urgent to seek the potential target to reduce the secretion of ECM or collagen synthesis and control liver fibrosis by inhibiting the activation of HSCs.

Bone marrow-derived mesenchymal stem cells (BM-MSCs) which are known to have plasticity, high proliferation, and differentiation capacity [6] possess an attractive option in the preclinical and clinical studies [7]. Moreover, multipotent MSCs can differentiate into numerous tissue lineages including hepatocytes [8]. Encouragingly, some clinical trials have proved that BMSCs can effectively alleviate end-stage liver disease and improve symptoms and liver function [9]. Additionally, human bone marrow-derived mesenchymal stem cells (hBM-MSCs) have the advantages of autologous sources and abundance of cells [10], suggesting being considered a promising tool for cell-based therapies of liver cirrhosis treatment. However, the underlying mechanisms are not yet well understood. Therefore, further exploration is required to better understand how MSCs regulate HSCs activation in the occurrence and development of liver cirrhosis.

In this study, hBM-MSCs were evaluated for their effectiveness in liver cirrhosis treatment by transplantation. We used mouse model of CCl_4_-induced liver cirrhosis to assess pathological effectiveness. We intended to investigate whether hBM-MSCs accommodate HSC activation and as well as possible repair mechanisms for liver fibrosis, focusing on its role in the signaling pathway mediating the effect of hBM-MSCs on HSC activation.

## 2. Materials and Methods

### 2.1. Reagents and Antibodies

Diphenyleneiodonium chloride (DPI, an inhibitor for NADPH oxidase signaling pathway), Cell Counting Kit-8, TRI-Reagent, and Hoechst 33342 were from Sigma (St. Louis, MO, USA), and Annexin V-FITC/PI Apoptosis Detection Kit were purchased from Beyotime (Jiangsu, China). Carbon tetrachloride (CCI_4_) (concentration 100%) and olive oil were obtained from Algomhoria Company. Enhanced BCA Protein Assay Kit was from Beyotime Biotechnology (Haimen, China). The primary antibodies including Bax, Bcl-2, cleaved caspase-3, *α*-smooth muscle actin (*α*-SMA), and *α*1(I) collagen were purchased from Abcam (Cambridge, MA, USA), and PPAR*γ*, p47phox (serine 359), p-p47phox, 4-hydroxynonenal (4-HNE), and *β*-actin were purchased from Santa Cruz (Santa Cruz, CA, USA).

### 2.2. Preparation of Human Bone Marrow-Derived Mesenchymal Stem Cells

Human bone marrow-derived mesenchymal stem cells were from healthy persons between 40 and 45 years of old who voluntarily donated bone marrow stem cells. Briefly, mesenchymal stem cells were collected into appropriately supplemented DMEM culture medium (Dulbecco's Modified Eagle's Medium, Gibco, San Diego, CA, USA), at 37°C/5% CO_2_ fully humidified atmosphere. The expressions of hBM-MSC surface markers cluster of differentiation (CD14, CD34, CD45, CD90, CD73, and CD105) were detected by BD FACS Calibur™ (BD Biosciences, San Jose, CA, USA), and cells were characterized as having high expression of these positive markers (CD90, CD73, and CD105) and low expression of the negative markers (CD14, CD34, and CD45) [11].

### 2.3. HSC Isolation and Culture

HSCs were isolated from adult Sprague-Dawley (SD) rats (8 weeks of age) or mice and primary rat HSCs were isolated using a two-step collagenase perfusion. Briefly, the rat liver was minced and incubated in 0.02% pronase with deoxyribonuclease (Sigma) after the rat liver perfused in situ with collagenase (Sigma) and streptavidin (Sigma). Then, the mixture was centrifuged to remove parenchymal cells and subsequently HSCs were recovered by density gradient centrifugation of nonparenchymal cells. HSCs were activated for 10–14 days by culturing in a 25 cm^2^ flask with DMEM (Gibco) containing 10% fetal bovine serum (Gibco) at 37°C/5% CO_2_ fully humidified atmosphere. The activated HSCs between passages 3 and 8 were used for experiments.

### 2.4. Coculture System

To evaluate the effects of the hBM-MSCs on activated HSCs, indirect coculture in Transwell chamber (24 mm diameter, 0.4 mm pore size; Corning) was used. The activated HSCs were mixed and cocultured with hBM-MSCs in supplemented DMEM containing 10% fetal bovine serum (FBS) at a 1 : 1 ratio for activated HSCs and hBM-MSCs. The activated HSC cells were inoculated in the lower part, and the hBMSCs were inoculated in the upper part. The cell numbers were evaluated using microscopy, and viability was determined by the trypan blue exclusion assay. As a comparison to the coculture system, the activated HSC group was seeded in the lower chamber and treated with or without 1 *μ*M DPI [12] or DMEM containing 10% FBS. Samples were collected after culturing for 24, 48, or 72 hours.

### 2.5. Cell Proliferation Assay and Apoptosis Analysis

Cell proliferation was determined using a sensitive colorimetric assay, the Cell Counting Kit-8 (CCK-8; Sigma-Aldrich, St. Louis, MO, USA). The coculture cells were collected after culturing for 24, 48, or 76 hours. The CCK-8 reagent was added to the lower chamber containing activated HSCs in the HSC group and the coculture group and incubated for 2 hours according to the manufacturer's protocol. For the apoptosis analysis, after culturing for 76 hours, the coculture cells were stained with Annexin V-FITC/PI Apoptosis Detection Kit by BD FACS Calibur (BD Biosciences, San Jose, CA, USA) and the test methods were implemented according to the manufacturer's instructions.

### 2.6. Assay of NADPH Oxidase Activity

NADPH oxidase (NOX) activity was measured using lucigenin as described elsewhere [13]. Briefly, the coculture cells or the activated HSCs were harvested after culturing for 24, 48, or 76 hours and centrifuged at 400 ×g for 10 min at 4°C, and the cell pellet was kept on ice after resuspended by 35 *μ*l/per well of the ice-cold RPMI-1640 medium. Then the NOX activity was measured in cell homogenates in a reaction mixture that contained NADPH (1 *μ*M) or lucigenin (20 *μ*M), 5 *μ*l of cell suspension, and a final 200 *μ*l volume of prewarmed (37°C) RPMI-1640 medium. The chemiluminescence was continuously recorded for 12 min, and the specific enzyme activity was calculated as counts per million cells.

### 2.7. Animals and Treatments

Male mice (10 to 12 weeks old, 20 to 28 g) were obtained from the animal center of the Central South University (Changsha, China). In brief, CCl_4_ was usually used for induction of mouse liver fibrosis [14]. Male mice were randomly separated into three groups (ten mice/each group): group 1 (olive oil + vehicle), group 2 (CCl_4_ + vehicle), and group 3 (CCl_4_ + hBM-MSCs). At first, the group 2 and group 3 were treated with CCl_4_ (diluted 1 : 5 in olive oil, 5 *μ*l/g) intraperitoneally twice weekly for 11 weeks. For the normal control group (group 1), the mice were injected with the same volume of olive oil.

After 11 weeks of CCl_4_ or olive oil administration, group 1 and group 2 received 0.1 mL PBS/mouse via the tail vein; group 3 was infused with 8 × 10^6^ hBM-MSCs in PBS/mouse via the liver portal vein. CCl_4_ administration was continued after hBM-MSC transplantation in accordance with earlier standards in case of spontaneous fibrosis recovery [15]. After 3 weeks of hBM-MSC transplantation, the liver was fixed in 4% buffered paraformaldehyde for immunostaining analysis or HSCs were isolated from mice for Western blot analysis. All animal experiments were performed in accordance with guidelines from the Central South University Institutional Animal Care and Use Committee.

### 2.8. Immunofluorescence Staining and Sirius Red Staining

For examination of the expressions of p47phox (a subunit of NADPH oxidase) and 4-hydroxynonenal (4-HNE) in HSCs in the mouse liver, a double fluorescent staining was performed as described elsewhere [12]. After being blocked with normal serum, tissue sections were incubated with primary rabbit anti-p47phox (1 : 100, Cat. SC-14015, Santa Cruz, CA, USA), rabbit anti-4-HNE (1 : 50, Cat. Ab46546, Abcam, Cambridge, MA, USA), and primary mouse anti-synaptophysin (SYP, 1 : 10, Cat. SC-365488, Santa Cruz, CA, USA), a marker for quiescent and activated HSCs [16]. The secondary antibodies, including DyLight 594-conjugated secondary antibody (1 : 500, Cat. GtxRb-003-D5948NHSX, ImmunoReagents Inc., Raleigh, USA) and DyLight 488-conjugated secondary antibody (1 : 500, Cat. GtxMu-003-D488NHSX, ImmunoReagents Inc., Raleigh, USA), were used according to the manufacturer's instructions. The nuclei were counterstained with Hoechst 33342. For single fluorescence staining of *α*-smooth muscle actin (*α*-SMA) on the liver sections, the paraformaldehyde-fixed liver sections were blocked with normal serum and then incubated with primary antibody against *α*-SMA (1 : 100) and the DyLight 594-conjugated secondary antibody, followed by counterstaining with the nuclear dye haematoxylin. Images were captured with the fluorescence microscope.

Sirius red staining of collagen was used to stain collagen on tissue sections for histological analysis of liver fibrosis. In short, paraformaldehyde-fixed liver tissue sections were incubated with picric acid-fast green (Amresco, Solon, USA), and then stained with picric acid Sirius red (Amresco, Solon, USA) for 1 hour. Images were captured by using a light microscope.

### 2.9. Measurement of Malondialdehyde, Glutathione Peroxidase, Superoxide Dismutase, Catalase, and Hydroxyproline in Liver Tissues

The malondialdehyde (MDA), glutathione (GSH), superoxide dismutase (SOD), and catalase (CAT) in liver tissues were analyzed according to the protocol provided by the commercial kits (Nanjing Jiancheng Bioengineering Institute, China). The results were shown as mmol/mg protein (MDA and GSH), U/mg protein (SOD and CAT), or mg/mg in wet liver (hydroxyproline).

### 2.10. Western Blot Analysis

The HSCs were harvested and centrifuged (10000*g*) at 4°C for 20 min to prepare the whole cell lysate. Next, by using an Enhanced BCA Protein Assay Kit according to the manufacturer's protocol. The supernatants were used for quantification of the total protein concentration. The denatured samples were analyzed by 10% SDS-PAGE gels and transferred onto nitrocellulose membrane. The blots were incubated with antibodies against Bax, Bcl-2, cleaved caspase-3, *α*-SMA, *α*1(I) collagen, p47phox, p-p47phox, NOX2, 4-HNE and PPAR*γ*, and subsequently by horseradish peroxidase-conjugated secondary antibody. *β*-Actin was used as an internal control.

### 2.11. RNA Isolation and Real-Time PCR

Total RNA was extracted from cells by using TRI-Reagent according to the manufacturer's instructions. Equal amounts of RNA were reverse transcribed to cDNA with a cDNA reverse transcription kit (Takara Biotechnology Co, Ltd.). Real-time PCR was performed with iTaqTM Universal SYBR Green (Bio-Rad Laboratories Inc., Hercules, CA, USA). The target gene was amplified according to the following conditions: initial denaturation at 94°C for 10 min; 35 cycles of denaturation at 94°C for 1 min, annealing at 55°C for 15 sec, and extension at 70°C for 15 sec; and a final extension at 70°C for 5 min.

The cycle threshold (Ct) values were normalized against the endogenous cyclophilin control and analyzed by using the ΔΔCt method [17]. The value was quantified by normalization to the cyclophilin mRNA level. Primers were listed in Table 1.

### 2.12. Statistical Analysis

The results are obtained as the mean ± standard deviation of at least three assays and the differences between means were evaluated by using an unpaired two-sided Student's *t*-test. *P* < 0.05 was considered as significant difference.

## 3. Results

### 3.1. Preparation of Human Bone Marrow-Derived Mesenchymal Stem Cells

**Figure 1:**
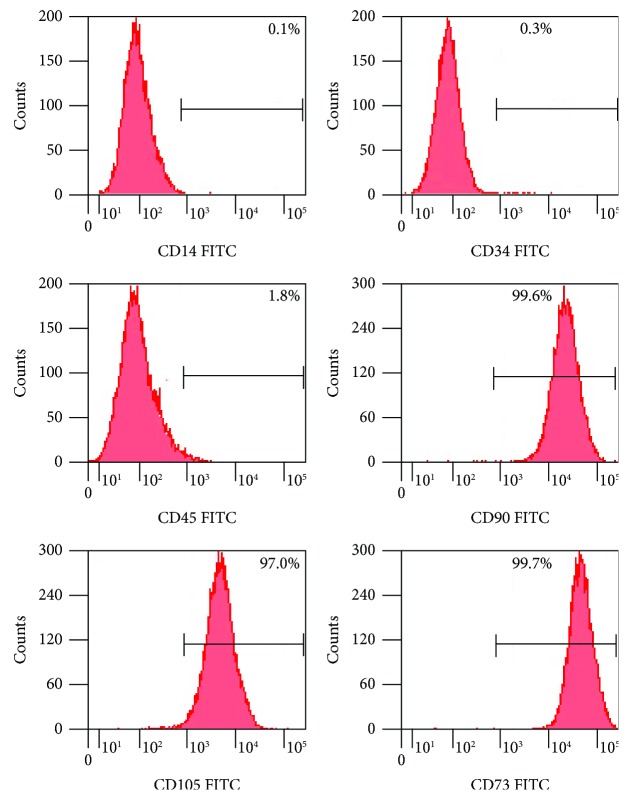
Preparation of human bone marrow-derived mesenchymal stem cells. The expressions of hBM-MSC surface markers' cluster of differentiation (CD14, CD34, CD45, CD90, CD73, and CD105) were detected by BD FACS Calibur.

The hBM-MSCs in culture are defined by the expression of cell surface markers as shown in Figure 1, we found that the hBM-MSCs exhibited strong expression of classical markers including, CD73, CD90 and CD105 expression and lack the expression of CD14, CD34, and CD45.The data presented here support that these hBM-MSCs in culture are consistent with the characteristics of stem cells [11].

### 3.2. The Inhibitory Effects of hBM-MSCs on Activated HSC Proliferation

**Figure 2:**
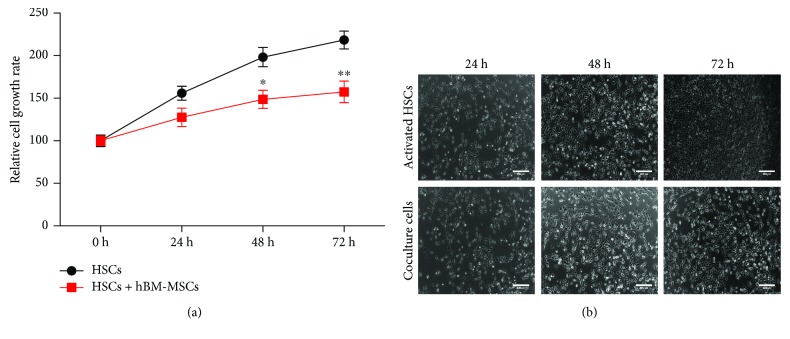
The inhibitory effects of hBM-MSCs on activated HSC proliferation. (a) The inhibitory effects of hBM-MSCs on activated HSC proliferation examined using CCK-8 assays in single cultures and cocultures at 24, 48, and 72 hours. Data are expressed as the mean ± standard deviation of three independent experiments. ^∗^*P* < 0.05 versus the control group and ^∗∗^*P* < 0.001 versus the control group. (b) Representative images of activated HSCs in single cultures and cocultures at 24, 48, and 72 hours. Scale bar = 200 *μ*m.

The Transwell coculture was used to determine the effects of hBM-MSC transplantation on the HSC proliferation; the amount of growing cells was visualized under an inverted microscope (Figure 2(a)). Next, to assess the effects of hBM-MSCs on the activated HSCs, as shown in Figure 2(b), CCK-8 assays were performed to measure the activated HSC proliferation, and there were no significant differences between single and cocultured activated HSCs at 24 hours; however, hBM-MSCs revealed the significant effect of suppressing the proliferation of activated HSCs at 48 hours and 72 hours when compared with those in the activated HSC group.

### 3.3. hBM-MSCs Induce Apoptosis Process in Activated HSCs

**Figure 3:**
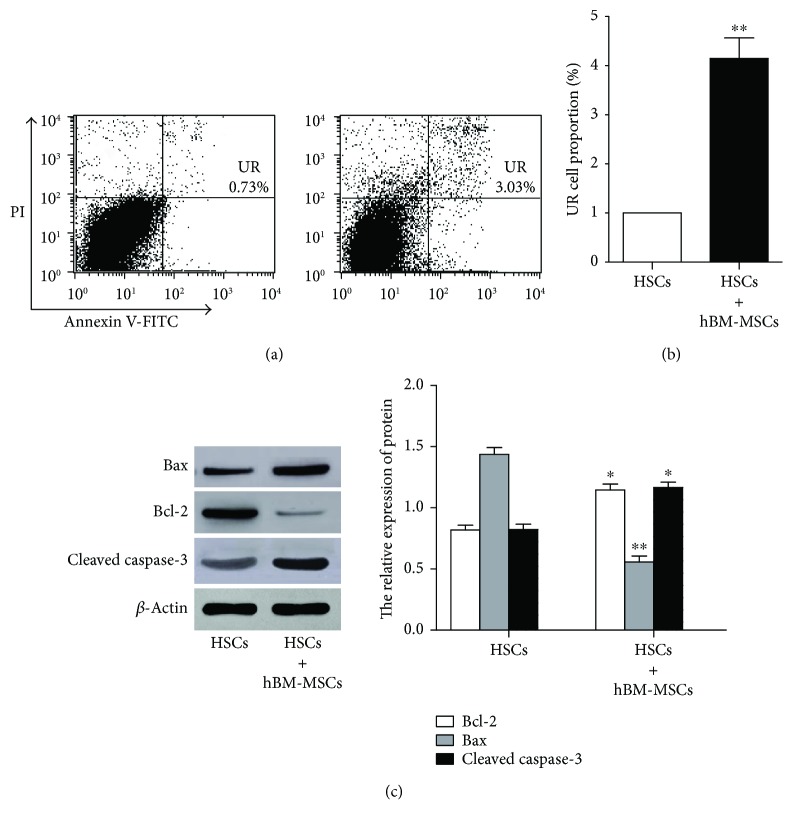
hBM-MSCs induce apoptosis process in activated HSCs. (a, b) The apoptosis of the cells was measured using flow cytometry in single cultures and cocultures at 72 hours. ^∗∗^*P* < 0.01 versus the control group. (c) The protein Bax, cleaved caspase-3, and Bcl-2 protein in single cultures and cocultures at 72 hours were, respectively, analyzed by Western blot analysis. ^∗^*P* < 0.05 versus the control group and ^∗∗^*P* < 0.001 versus the control group.

Since we have confirmed that the inhibitory effects of hBM-MSCs on activated HSC proliferation, next, we explored whether the antiproliferative activity of hBM-MSCs is related to apoptosis. The flow cytometry was used after double staining with Annexin V-FITC/PI to determine the cell apoptosis. The cells of coculture group and the activated HSC group were collected after culturing for 48 hours, and then Figures 3(a) and 3(b) suggested that the apoptotic cell number of activated HSCs in the coculture group was significantly increased at 48 hours when compared with that in the activated HSC group at 48 hours. Furthermore, we also investigated the expression of Bcl-2, Bax, and cleaved caspase-3 [18] to investigate the underlying mechanism of the apoptosis process of hBM-MSCs on activated HSCs. The proteins Bcl-2, Bax, and cleaved caspase-3 in single cultures and cocultures at 48 hours were tested by Western blot analysis (Figure 3(c)). The results showed in the coculture system protein that the signal of antiapoptotic protein Bcl-2 was weakened. In contrast, the proapoptotic proteins Bax and cleaved caspase-3 protein Bax were gradually increased.

### 3.4. NADPH Oxidase Pathway Mediates hBM-MSC Inhibition of HSC Activation

We previously determined that hBM-MSCs resulted in the loss of activated HSC proliferation and induced the apoptosis of activated HSCs. However, the detailed mechanism involved in this phenomenon remains unclear. Accumulating evidence indicates ROS are critical intermediates in liver physiology and pathology [19]. Moreover, the NADPH oxidase (NOX) family, which acts as a predominant mediator of redox homeostasis, may contribute to ROS production during liver fibrosis [20]. Thus, we attempted to investigate the roles of NADPH oxidase involved in hBM-MSC regulation of activated HSCs.

**Figure 4:**
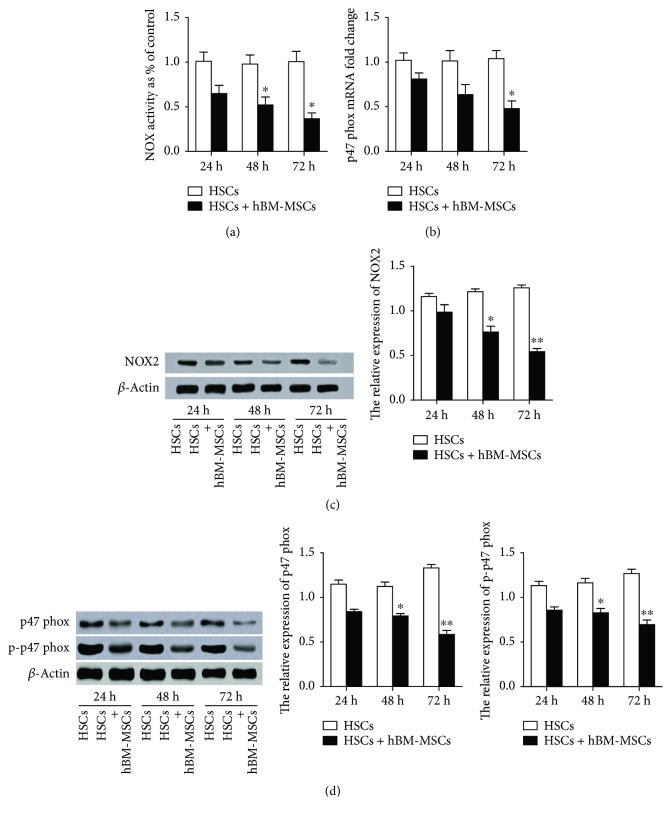
NADPH oxidase pathway mediates hBM-MSC inhibition of HSC activation. The cocultures and activated HSCs were harvested after being cultured for 24, 48, and 72 hours. (a) NADPH oxidase activity was measured. (b) p47phox mRNA level relative to cyclophilin was analyzed by real-time PCR (*n* = 3). ^∗^*P* < 0.05 versus the control group. (c, d) NOX2, p47phox, and p-p47phox were evaluated by Western blot. Experiments were performed in triplicate. ^∗^*P* < 0.05 versus the control group and ^∗∗^*P* < 0.001 versus the control group.

The coculture cells or the activated HSCs were cultured for 24, 48, and 72 h; Figure 4(a) showed that the effects of hBM-MSCs reduced the NOX activity in a time-dependent manner, suggesting that the pathways of NADPH oxidase might contribute the effect of hBM-MSCs on HSC activation.

The functional active component of the NADPH oxidase complex, p47phox, is considered to play a central role in the activity of NADPH oxidase and the regulation of HSC activity and liver fibrosis [21]. Thereby, p47phox was used to examine the respective signaling pathway. As shown in Figure 4(b), the coculture cells revealed a significant decrease in p47phox mRNA level as compared with the control. As expected in Figures 4(c) and 4(d), hBM-MSCs caused the decrease in the levels of NADPH oxidase transmembrane subunit NOX2 and its ligand p47phox in a time-dependent manner on HSC activation. Considering that p47phox phosphorylation regulates activation of NADPH oxidase [22], we examined the phosphorylation of p47phox. This approach revealed that the serine 359 phosphorylation of the p47phox subunits was significantly decreased with the treatment of hBM-MSCs compared with the control group. This finding encouraged us to speculate that NADPH oxidase was required for the inhibition effect of hBM-MSCs on HSC activation.

### 3.5. The Pathways of NADPH Oxidase Is Involved in hBM-MSC-Induced Reduction in Liver Fibrosis

**Figure 5:**
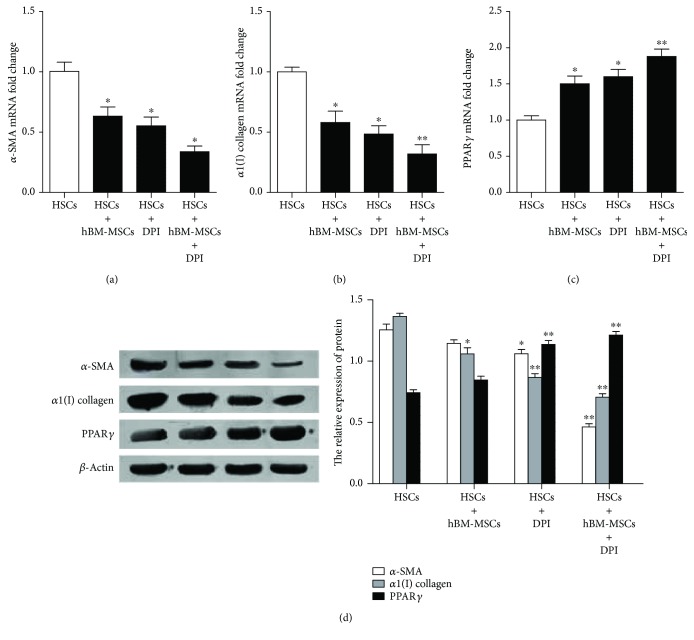
The pathways of NADPH oxidase are involved in hBM-MSC-induced reduction in liver fibrosis. The cocultures and activated HSCs were pretreated with or without 1 *μ*M DPI for 1 hour, and then cells were harvested after cultured for 72 hours. (a–c) The mRNA levels of PPAR*γ*, *α*-SMA, and *α*1(I) collagen were, respectively, detected by real-time PCR (*n* = 3). ^∗^*P* < 0.05 versus the control group. ^∗∗^*P* < 0.001 versus the control group. ^∗^*P* < 0.05 versus activated HSCs plus DPI. (d) The proteins of PPAR*γ*, *α*-SMA, and *α*1(I) collagen were evaluated by Western blot. ^∗^*P* < 0.05 versus the control group. ^∗∗^*P* < 0.001 versus the control group.

The signaling pathways of NADPH oxidase were suggested to mediate hBM-MSC reduction of HSCs; thereby, to evaluate the potential contribution of NADPH oxidase in liver fibrosis, the expression of Peroxisome proliferator-activated receptor *γ* (PPAR*γ*) (a hallmark of adipocytes), *α*-SMA, and *α*1(I) collagen (two markers of activated HSCs) was tested. After the activated HSCs or the cocultured were pretreated with 1 *μ*M DPI (an inhibitor for NADPH oxidase signaling pathway), the mRNA and protein levels of PPAR*γ*, *α*-SMA, and *α*1(I) collagen were detected. The results in Figure 5 showed that after the activated HSC incubation with DPI or with hBM-MSCs, the mRNA and protein expression of *α*-SMA and *α*1(I) collagen decreased, whereas the expression of PPAR*γ*, considerably increased. What is more, the inhibitor for NADPH oxidase signaling pathway strengthened the hBM-MSCs, the promotion effect on PPAR*γ* and inhibition of *α*1(1) procollagen and *α*-SMA expressions. These consistent findings in two different cell treatment supported the critical role of NADPH oxidase and hBM-MSCs in regulating HSC activation.

### 3.6. hBM-MSCs Lead to the Decrease in Liver Fibrosis in the Mouse Model of CCl_4_-Induced Liver Injury

**Figure 6:**
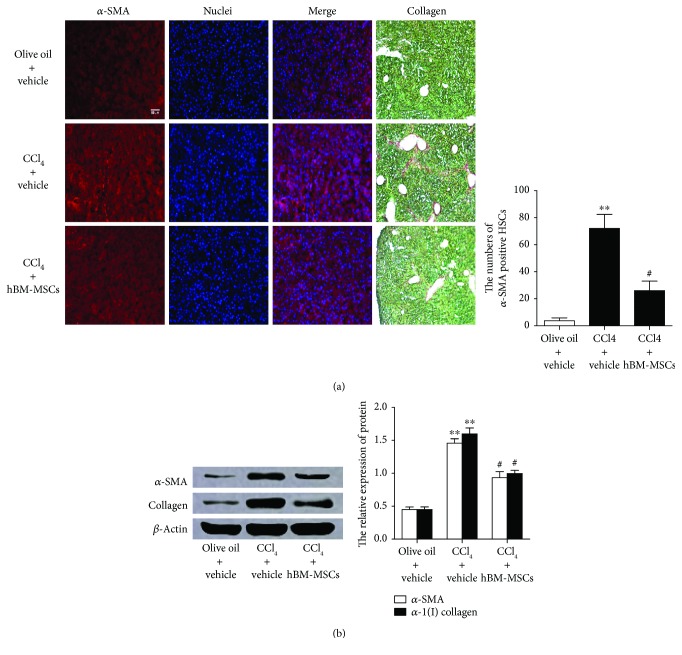
hBM-MSCs lead to the decrease in liver fibrosis in the mouse model of CCI_4_-induced liver injury. Mice were divided into three groups and were, respectively, given administration of olive oil plus vehicle, CCI_4_ (5 *μ*l/g body weight, two times a week) plus vehicle or CCI_4_ (5 *μ*l/g body weight, two times a week) plus hBM-MSCs (8 × 10^6^/mouse). (a) Single fluorescence staining of *α*-SMA on the liver sections was detected (red fluorescence); the nuclei (blue fluorescence) were counterstained with Hoechst 33342. The images were captured with the fluorescence microscope. Scale bar = 100 *μ*m. And stain collagen was examined by Sirius red staining of collagen. The representative images were captured with a light microscope. Scale bar = 50 *μ*m. The number of *α*-SMA-positive HSCs in six randomly chosen fields was counted at 100-fold magnification, and the average values were shown. ^∗∗^*P* < 0.001 versus the control group. ^#^*P* < 0.05 versus the mice of group treatment with CCI_4_ plus vehicle. (b) The protein levels of *α*-SMA and *α*1(I) collagen were examined by Western blot analysis after the HSCs were isolated from each group. ^∗∗^*P* < 0.001 versus the control group. ^#^*P* < 0.05 versus the mice of group treatment with CCI_4_ plus vehicle.

Based on the above results, to further investigate whether hBM-MSCs influenced liver fibrosis in vivo, we examined the expressions of *α*-SMA and collagen in vivo. CCl_4_ can induce liver fibrosis in normal mice, and this is a well-established model of liver injury [14]. Briefly, mice were randomly separated into three groups: group 1 (olive oil + vehicle), group 2 (CCl_4_ + vehicle), and group 3 (CCl_4_ + hBM-MSCs). Almost all the mice develop liver fibrosis throughout the 11-week period of CCl_4_ treatment, three groups of mice were coadministered, with or without hBM-MSCs. Next, the *α*-SMA-positive cells in the livers were detected by immunofluorescence to evaluate the HSC activation and the collagen stained by Sirius red was examined to appraise liver fibrosis.  Figure 6(a) shows that *α*-SMA-positive cells or the signs of liver fibrosis were barely detectable in group 1 (olive oil + vehicle) whereas HSC activation (the number of *α*-SMA-positive cells) and liver fibrosis sharply increased in group 2 (CCl_4_ + vehicle). However, when mice in group 3 received hBM-MSCs, the promoting effect of CCl_4_ was suppressed and the *α*-SMA-positive cells and liver fibrosis were partially suppressed. Western blot analysis further proved the protective effect of hBM-MSCs on liver fibrosis. We isolated the HSCs from groups 1–3 and examined the protein levels of *α*-SMA and *α*1(I) collagen. The results (Figure 6(b)) revealed that, compared with group 1, CCl_4_ increased the expressions of *α*-SMA and *α*1(I) collagen (group 2), and these effects were attenuated by the hBM-MSC transplantation (group 3). Accordingly, the extent of liver fibrosis changed in concordance with the alteration of HSC activation.

Collectively, these in vivo observations provide further support to the role of hBM-MSCs in HSC activation and liver fibrogenesis.

### 3.7. hBM-MSCs Decrease the Levels of 4-HNE and p47phox in HSCs in Mouse Model of CCl_4_-Induced Liver Fibrosis

**Figure 7:**
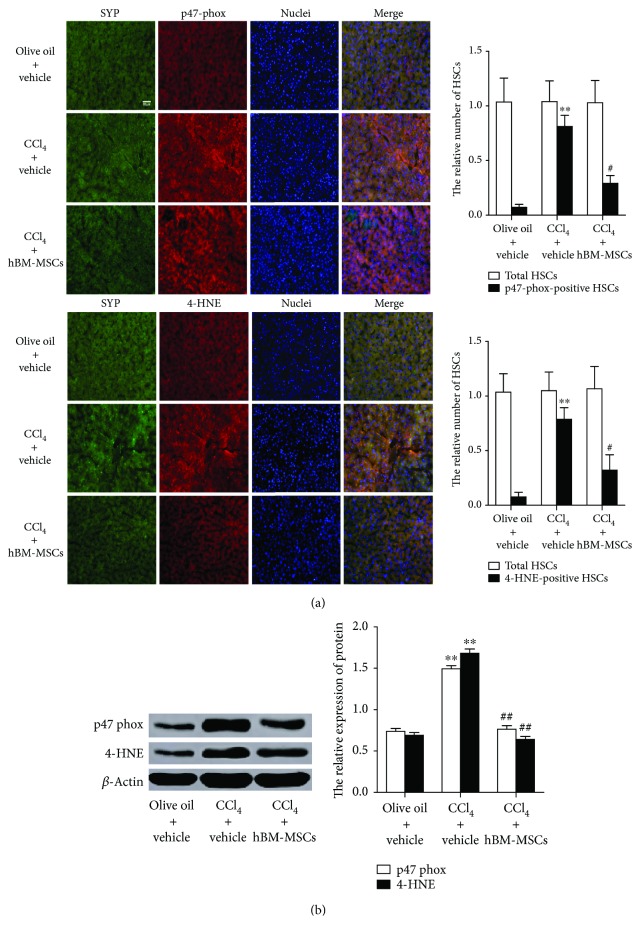
hBM-MSCs decrease the levels of 4-HNE and p47phox in HSCs in a mouse model of CCl_4_-induced liver fibrosis. As described in Figure 6, male mice were randomly separated into three groups; the mice of group 1 served as normal control group, and the other two groups of mice, respectively, received hBM-MSCs (8 × 10^6^/mouse) or vehicle throughout the 11-week period of CCI_4_ (5 *μ*l/g body weight, two times a week). (a) Double-staining was performed on liver section: synaptophysin (SYP, a marker for quiescent and activated HSCs) (green fluorescence); 4-HNE and p47phox (markers for positive HSCs) (red fluorescence); nuclei (blue fluorescence). The images were captured with the fluorescence microscope. Scale bar = 50 *μ*m. The total HSCs (SYP-positive HSCs) and the relative number of 4-HNE and p47phox-positive HSCs in six randomly chosen fields were counted at 100-fold magnification, and the average values were shown. ^∗∗^*P* < 0.001 versus the control group. ^#^*P* < 0.05 versus the mice of group treatment with CCI_4_ plus vehicle. (b) The protein levels of 4-HNE and p47phox were examined by Western blot analysis after the HSCs were isolated from each group. ^∗∗^*P* < 0.001 versus the control group. ^##^*P* < 0.001 versus the mice of group treatment with CCI_4_ plus vehicle.

We have shown that hBM-MSCs can restrain NADPH oxidase signaling pathway and reduced liver fibrosis in HSCs in mouse model of CCl_4_-induced liver injury; the role of this pathway in mouse model of CCl_4_-induced liver fibrosis was further investigated. To assess whether hBM-MSCs influenced NADPH oxidase in the same model in vivo, we examined the productions of 4-HNE (a lipid peroxidation product as a general marker of ROS) and p47phox in HSCs. The mice were given the administration of vehicle or hBM-MSCs throughout the 11-week period of CCl_4_ treatment, the positive HSCs for 4-HNE and p47phox in livers were examined by double fluorescence staining. SYP can be used as a marker for quiescent and activated HSCs [23].  Figure 7(a) showed representative photomicrographs of immunofluorescent analysis for 4-HNE and p47phox (red colour) and SYP (green colour) in the liver sections from each group. Double fluorescent staining the relative number of 4-HNE and p47phox-positive HSCs demonstrated that, compared with group 1 (olive oil + vehicle), CCl_4_ increased 4-HNE and p47phox-positive HSCs in group 2 (CCl_4_ + vehicle), which was partially counteracted by hBM-MSC transplantation in group 3 (CCl_4_ + hBM-MSCs), which suggested the inhibitory role of hBM-MSCs in inductions of 4-HNE and p47phox in HSCs in the model.

To confirm the above results, HSCs were isolated from each group and directly used for detecting 4-HNE and p47phox protein levels by Western blot analysis (Figure 7(b)). As expected, CCl_4_ clearly increased the 4-HNE and p47phox protein level. These results further support the role of the NADPH oxidase pathway in the inhibitory effects of hBM-MSCs on HSCs in vivo.

### 3.8. Effects of hBM-MSCs on MDA, GSH, SOD, and CAT Level in Liver Tissues

**Figure 8:**
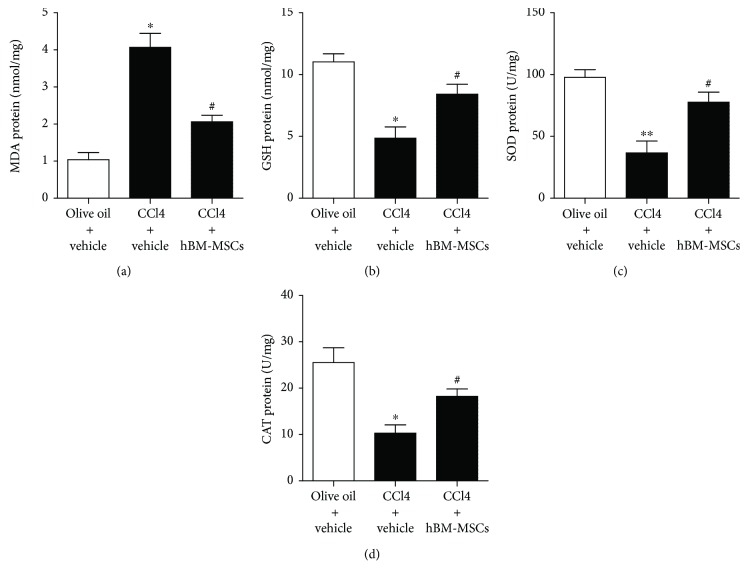
Effects of hBM-MSCs on MDA, GSH, SOD, and CAT level in liver tissues. The levels of MDA, GSH, SOD, and CAT in liver tissues in the three groups were detected with chromatometry. ^∗^*P* < 0.05 versus the control group. ^∗∗^*P* < 0.001 versus the control group. ^#^*P* < 0.05 versus the mice of group treatment with CCI_4_ plus vehicle.

Reports have indicated that liver injury can improve oxidative stress through regulating glutathione (GSH) metabolism and attenuating oxidative damage to lipids and proteins, resulting in an antioxidative response [24]. In the present study, in Figure 8, CCl_4_ injection markedly increased malondialdehyde (MDA) level and decreased GSH, superoxide dismutase (SOD), and catalase (CAT) levels, whereas hBM-MSCs significantly protected the liver against this effect.

## 4. Discussion

Hepatic fibrosis is known to be a common clinical symptom. The critical role of HSC activation in early development has been previously found in liver fibrosis [25] In recent years, the mesenchymal stem cells (MSCs) due to their practical advantages in regenerative medicine have received more and more attention. The bone marrow-derived- (BM-) MSCs are readily obtained and have favorable characteristics including ease of handling in vitro, self-renewal, multipotent differentiation, and low immunogenicity [26]. Given that the inhibition of the activated HSCs is the central event of the reversal of hepatic fibrosis and BM-MSCs are the excellent clinical candidates in the treatment of hepatic fibrosis, this study has been concerned on establishing proofs for seeking the molecular mechanisms underlying hBM-MSC inhibition of HSCs activation and thus relieving the pathological liver fibrosis in vitro and vivo. These data focus on talking over three aspects: (1) the hBM-MSCs inhibit HSC proliferation and induce apoptosis process in activated HSCs; (2) the positive effect of the hBM-MSCs-downregulated NADPH oxidase pathway influences the levels of PPAR*γ*, *α*-SMA, and *α*1(I) collagen in vitro; (3) and the hBM-MSCs play an important role in protecting CCI_4_-induced liver fibrosis via inhibition of the NADPH oxidase pathway.

The BM-MSCs could reduce the proliferative capability of activated HSCs and have the potential to act as a proliferative function through changing cell-cycle distribution [27]. The beneficial effects of MSCs on liver fibrosis may be mediated by autocrine and paracrine mechanisms. It is reported that BMSCs can enhance the apoptosis of activated HSCs through secreting paracrine factors [28]. In fact, our report indicated that hBM-MSCs inhibited the proliferation of activated HSCs by inducing the apoptosis process of activated HSCs. In this regard, the BM-MSCs exerted a promoting effect in apoptosis process of activated HSCs by two-cell apoptotic proteins, antiapoptotic protein Bcl-2, and proapoptotic protein Bax [18]. The Bcl-2/Bax signaling is the key to mitochondrial-mediated pathway and an important regulator of the intrinsic apoptosis [29]. Importantly, the results were consistent with those of other antifibrotic drugs [30], which have been shown to promote the apoptosis of activated HSCs by downregulating Bcl-2 and upregulating Bax and cleaved caspase-3.

In addition, multiple signaling pathways are involved in the effect of the MSC-induced HSC growth inhibition [31]. We noticed that the generation of reactive oxygen species (ROS) is interrelated and interacts on hepatic fibrosis processes, and NADPH oxidases (NOX) work as a key source of ROS [32]. What is more, NADPH oxidase signaling pathway is closely related in experimental models of liver fibrosis and in patients with chronic HCV-derived infection [33]. There was a possibility that a potentially effective mechanisms between hBM-MSCs and HSCs might exist. In in vitro experiments, hBM-MSCs would significantly reduce the NOX activity. p47phox, a functional active component of the NADPH oxidase complex, is considered to play a crucial role in the activity of NADPH oxidase [20]. In our study, a slight reduction in p47phox expression had been found. Interestingly, the phosphorylation of the p47phox subunits was significantly decreased by the effect of the hBM-MSCs. Thus, the results that the hBM-MSCs may restrain the NADPH oxidase activation by phosphorylation of p47phox may a better understanding of the inhibition effect of hBM-MSCs on NADPH oxidase signaling pathway.

The accumulation of extracellular matrix ECM which is mainly produced by activated HSCs is one of the important characteristics for the reaction of fibrosis [3]. Peroxisome proliferator-activated receptor *γ* (PPAR*γ*) exerts a key role in the inhibition of HSC activation [34]. Our researches showed that using either DPI (an inhibitor for NADPH oxidase pathway) or the hBM-MSCs would result in a dramatic drop of *α*SMA (a well-established marker for HSC activation) and *α*(I) collagen (the major component of ECM) in activated HSCs in vitro and a rocket increasing of the PPAR*γ* expression. The inhibitor for NADPH oxidase pathway, DPI, significantly strengthened the hBM-MSCs, the promotion effect on PPAR*γ*, and inhibition of *α*1(1) collagen and *α*-SMA expressions. This result suggested that hBM-MSCs might regulate activated HSCs expression, at least in part, by inhibiting the NADPH oxidase pathway.

Multiple signaling pathways or channels have been confirmed to participate in the regulatory mechanism of hBM-MSCs on HSC activation [35, 36], and our study may provide a new clue and possibility for explaining the protective effect of hBM-MSCs on liver fibrosis. The antifibrotic effects of the hBM-MSCs were also investigated by carbon tetrachloride- (CCl_4_-) induced liver fibrotic mouse model via the liver portal vein; the hBM-MSCs recover liver function markers at 21 days after transplantation.

Our results revealed that NADPH oxidase was required for hBM-MSC-induced decrease in HSC activation in vitro and in vivo. 4-Hydroxynonenal (HNE) mediates oxidative stress-linked pathological processes [27], and a large number of positive HSCs for 4-HNE and p47phox was tested in mouse cirrhosis liver [12]. As we have demonstrated, CCl_4_ increased 4-HNE and p47phox-positive HSCs whereas the expression of 4-HNE and p47phox-positive HSCs was reduced with the treatment of hBM-MSCs. In combination with the above in vivo experiments, hBM-MSCs deduce the NADPH oxidase pathway, accompanied by the inhibitions of HSC activation and liver fibrosis in CCl_4_-induced liver injury of mice. The stimulation of GSH synthesis and the inhibition of GSH depletion are major contributors to the antioxidative mechanism in liver injury. In our study, CCl_4_ injection markedly decreased GSH levels, whereas hBM-MSCs significantly reversed this effect.

In summary, we have shown that hBM-MSCs are able to suppress the HSC activation and hBM-MSCs were shown to induce the apoptosis of activated HSCs. Furthermore, in vitro and in vivo, we showed that the antifibrotic effect of hBMSCs was mediated at least through the signaling pathways of NADPH oxidase. These data suggested that hBMSCs play an important role in protecting CCI_4_-induced HSC activation via inhibition of the CCI_4_ NADPH oxidase pathway and may represent a novel therapeutic way for the disease. These findings here not only greatly clarified the mechanism of hepatic fibrosis and provided a novel treatment option for the liver fibrosis but also made a thorough inquiry into the preclinical mechanisms of MSC's regenerative medicine.

## Figures and Tables

**Table 1:** Sequences of DNA primers for real-time PCR in this study.

Gene	Primer sequences
Rat p47phox	Forward 5′-CAGCCATGGGGGACACCTTCATT-3′
	Reverse 5′-GCCTCAATGGGGAACATCTCCTTCA-3′
Rat *α*-SMA	Forward 5′-ACAACGTGCCTATCTATGAGGGCT-3′
	Reverse ′-AGCGACATAGCACAGCTTCTCCTT-3′
Rat *α*1(I) collagen	Forward 5′-TGGTCCCAAAGGTTCTCCTGGT-3′
	Reverse 5′-TTAGGTCCAGGGAATCCCATCACA-3′
Rat cyclophilin	Forward 5′-TGGATGGCAAGCATGTGGTCTTTG-3′
	Reverse 5′-CTTCTTGCTGGTCTTGCCATTCCT-3′
